# Initial Experience in CT-Guided Percutaneous Transthoracic Needle Biopsy of Lung Lesions Performed by a Pulmonologist

**DOI:** 10.3390/jcm8060821

**Published:** 2019-06-08

**Authors:** June Hong Ahn, Jong Geol Jang

**Affiliations:** Department of Internal Medicine, Yeungnam University Medical Center, College of Medicine, Yeungnam University, Daegu 42415, Korea; jonggirl83@naver.com

**Keywords:** CT, percutaneous transthoracic needle biopsy, pulmonologist

## Abstract

In the diagnosis of lung lesions, computed tomography (CT)-guided percutaneous transthoracic needle biopsy (PTNB) has a high diagnostic yield and a low complication rate. The procedure is usually performed by interventional radiologists, but the diagnostic yield and safety of CT-guided PTNB when performed by pulmonologists have not been evaluated. A retrospective study of 239 patients who underwent CT-guided PTNB at Yeungnam University Hospital between March 2017 and April 2018 was conducted. A pulmonologist performed the procedure using a co-axial technique with a 20-gauge needle. Then diagnostic yield and safety were assessed. The overall sensitivity, specificity, positive predictive value, and negative predictive value for the diagnosis of malignancy were 96.1% (171/178), 100% (46/46), 100% (171/171), and 86.8% (46/53), respectively. The diagnostic accuracy was 96.9% (217/224) and the overall complication rate was 33.1% (82/248). Pneumothorax, hemoptysis, and hemothorax occurred in 27.0% (67/248), 5.2% (13/248), and 0.8% (2/248) of the patients, respectively. Univariate analyses revealed that pneumothorax requiring chest tube insertion was a significant risk factor (odds ratio, 25.0; *p* < 0.001) for diagnostic failure. CT-guided PTNB is a safe procedure with a high diagnostic accuracy, even when performed by an inexperienced pulmonologist. The results were similar to those achieved by interventional radiologists as reported in previously published studies.

## 1. Introduction

In the work-up of lung lesions, percutaneous transthoracic needle biopsy (PTNB) is a well-established procedure with a high diagnostic yield and an acceptable complication rate [1,2,3]. Compared to traditional flexible bronchoscopy, PTNB is useful for diagnosing peripheral lung lesions. Although pulmonologists are well versed in the use of virtual bronchoscopy, electromagnetic navigation bronchoscopy, and radial probe endobronchial ultrasound, their experience in performing PTNB is limited, despite a diagnostic yield of >90% for the procedure [4].

Computed tomography (CT)-guided PTNB was first described in 1976 [5]. Since then, numerous reports have shown that, in the diagnosis of peripheral lung lesions, it is an accurate procedure with a low complication rate [6,7,8,9,10,11]. Although there are several studies on the feasibility and safety of ultrasound-guided transthoracic biopsy performed by pulmonologists [12,13,14,15], CT-guided PTNB is typically conducted by interventional radiologists, and the experience of interventional pulmonologists has not been documented. Thus, we assessed the diagnostic yield, safety, and factors affecting the yield of CT-guided PTNB performed by a pulmonologist in routine clinical practice.

## 2. Methods

This was a retrospective study of 239 patients who underwent CT-guided PTNB at Yeungnam University Hospital (a 930-bed, university-affiliated, tertiary referral hospital in Daegu, South Korea). The study was conducted in accordance with the Declaration of Helsinki and was reviewed and approved by the Institutional Review Board of Yeungnam University Hospital (IRB number YUMC IRB 2018-11-019). The requirement for informed consent was waived because of the study’s retrospective design.

### 2.1. Patients

**Figure 1:**
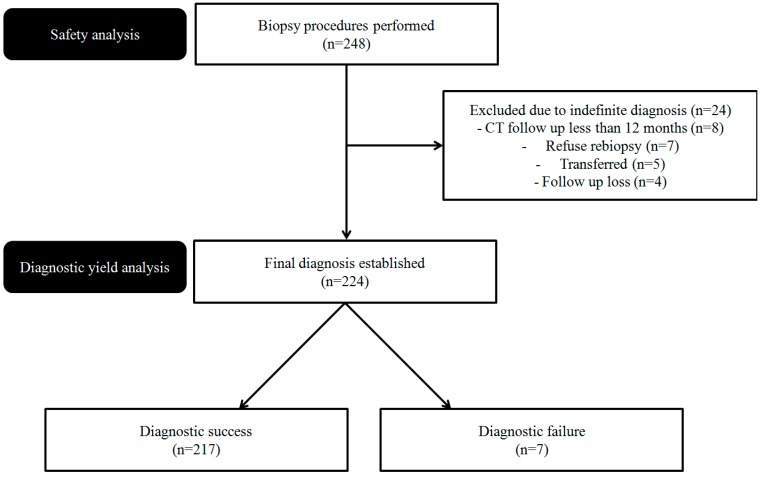
Study flowchart.

From March 2017 to April 2018, 248 CT-guided PTNB procedures were performed at our institution in 239 patients with lung lesions. Of these, 24 were excluded from diagnostic yield analyses for the following reasons: indefinite diagnosis, defined as a lesion with nonspecific benign features of stable or indeterminate size and a follow-up of <12 months (*n* = 8); refusal of re-biopsy for definite diagnosis (*n* = 7); transfer of the patient to another hospital for re-biopsy (*n* = 5); and lost to follow-up (*n* = 4). Ultimately, data from 224 lung lesions were included in the analyses (Figure 1). Because of nonspecific biopsy results, re-biopsy was performed in six patients.

### 2.2. Biopsy Procedure

CT-guided PTNBs were performed by one pulmonologist (J.H.A) with 3 years of experience in respiratory medicine and no experience in CT-guided PTNB. All procedures were conducted under CT guidance (Siemens SOMATOM Definition AS 64-slice computed tomography system, Siemens Healthcare, Erlangen, Germany). Before the procedure, the patient was instructed regarding the need for a breath-hold during inspiration or expiration as appropriate. The patient was placed in the supine, prone, or decubitus position depending on the location of the lesion, and a reference needle was placed near the puncture site. CT scanning was performed with a slice thickness of 2.4 mm. The pulmonologist determined the best needle trajectory to target the lung lesion, considering both the need for diagnostic accuracy and the complications associated with PTNB. After sterilization of the puncture site using betadine, local anesthesia consisting of 2% lidocaine was administered at the needle entry site. A coaxial introducer was inserted into the target lesion, and then a CT scan was performed to identify the exact location of the needle tip within the target lesion. If the coaxial needle tip was located correctly, a 20-gauge cutting needle (Stericut; TSK Laboratory, Tochigi, Japan) was inserted into the target lesion via the coaxial introducer, and biopsy was performed. If the specimen was inadequate, repeat specimens were obtained without additional pleural puncture. The specimens were fixed in 10% formalin for pathologic examination. An immediate post-procedural CT scan was performed to identify biopsy-related complications.

### 2.3. Diagnostic Performance

A final diagnosis of malignancy was made based on the following criteria: definite histological evidence of malignancy (*n* = 130), surgical confirmation of malignancy (*n* = 42), confirmation of malignancy by repeat biopsy (*n* = 4), confirmation of malignancy by bronchoscopic biopsy (*n* = 1), and clinical features consistent with malignancy together with increased tumor marker levels and typical positron emission tomography-CT results (*n* = 1). The lung lesion was diagnosed as benign according to the following criteria: Identification of definite benign features (*n* = 33), such as chronic granulomatous inflammation with caseous necrosis suspicious of tuberculosis, organizing pneumonia, tuberculous granuloma, pulmonary cryptococcosis, progressive massive fibrosis, pulmonary infarction associated with pulmonary embolism, or granulomatosis with polyangiitis; regression of the lesion with medical treatment (*n* = 10); surgical confirmation of a benign lesion (*n* = 2); and a stable size for at least 12 months (*n* = 1). All lung lesions with an indefinite diagnosis (neither benign nor malignant) were defined as indeterminate at the final diagnosis and excluded from the diagnostic yield analyses.

### 2.4. Statistical Analyses

To determine the risk factors for diagnostic failure, the study population was divided into two groups: a diagnostic success group (true-positive and true-negative results) and a diagnostic failure group (technical failure, false-positive and false-negative results). Lesions with indeterminate results and thus an indefinite diagnosis were excluded from the diagnostic yield analyses. Continuous variables were compared using Student’s *t*-test or the Mann-Whitney U-test and are expressed as the mean ± standard deviation (SD). Categorical variables were compared using the chi-square test or Fisher’s exact test. In all analyses, a *p* value < 0.05 was considered to indicate statistical significance based on two-tailed tests. All statistical procedures were performed using SPSS software (version 21.0; SPSS Inc., Chicago, IL, USA).

## 3. Results

### 3.1. Characteristics of the Patients, Lung Lesions, and Procedures

The baseline characteristics of the patients, lung lesions, and procedures are summarized in Table 1 (*n* = 248). The mean age of the patients was 68.2 ± 12.5 years, and 70.2% (*n* = 174) were male. The anatomical distribution of the lung lesions was as follows: 59.3% (*n* = 147) in the upper lobes, 37.1% (*n* = 92) in the lower lobes, and 3.6% (*n* = 9) in the middle lobes. Most of the lesions (*n* = 228, 91.9%) were solid, followed by subsolid (*n* = 17, 6.9%), and ground glass opacity (*n* = 3, 1.2%), respectively. The mean diameter of the lesions was 39.5 ± 19.3 mm, and 12.5% (*n* = 31) were cavitary lesions.

During the procedures, 64 (25.8%) patients were placed in the supine position, 141 (56.9%) in the prone position, and 43 (17.3%) in the decubitus position. All procedures were conducted using a 20-gauge needle. The mean number of specimens per procedure was 1.7 ± 0.7, and the mean length of the aerated lung traversed by the needle was 14.5 ± 15.3 mm. In 30 (12.1%) patients, emphysema along the needle pathway was detected. Twenty-six (10.5%) patients cooperated poorly with the respiration instructions. A transfissural approach was performed in 13 (5.2%) patients.

### 3.2. Pathologic Results and Diagnostic Accuracy

The initial pathologic results and final diagnosis are summarized in Table 2. The diagnosis in 178 (71.8%) of the 248 lung lesions was malignant, in 46 (18.5%) it was benign, and in 24 (9.7%) it was indeterminate. Of the 178 malignant lesions, 171 (96.1%) were diagnosed by PTNB. False-negatives (*n* = 3) and technical failures (*n* = 4) were diagnosed as malignancy by repeat PTNB (*n* = 4), bronchoscopic biopsy (*n* = 1), surgical resection (*n* = 1), or increased tumor marker levels and typical PET-CT results (*n* = 1). Of the benign lesions (*n* = 46), 33 were confirmed based on the detection of definite benign features, including by PTNB in 31 lesions, the surgical resection specimen in 1 lesion, and by sputum culture in 1 lesion. Pulmonary tuberculosis (*n* = 14, 5.6%) was the most common diagnosis among the definite benign cases. The diagnostic yield of CT-guided PNTB is shown in Table 3. Excluding the indeterminate cases, the sensitivity, specificity, positive predictive value (PPV), negative predictive value (NPV), and diagnostic accuracy of CT-guided PTNB were 96.1% (171/178), 100% (46/46), 100% (171/171), 86.8% (46/53), and 96.9% (217/224), respectively.

### 3.3. PTNB-Related Complications

The complications associated with the procedure are summarized in Table 4. The overall complication rate in our study was 33.1% (82/248). Pneumothorax occurred in 27.0% (67/248), and chest tube insertion was needed in 6.5% (16/248) of the patients. Hemoptysis developed in 5.2% (13/248) but the patients recovered after oxygen therapy and close observation. Hemothorax occurred in 0.8% (2/248) and was managed in all patients by chest tube insertion.

### 3.4. Risk Factors for Diagnostic Failure

The diagnostic success group (*n* = 217) consisted of 171 true-positive results and 46 true-negative results. The diagnostic failure group (*n* = 7) consisted of three false-negative results and four technical failures.

The results of univariate analyses are shown in Table 5. Pneumothorax requiring chest tube insertion was the only significant risk factor (odds ratio, 25.0; *p* < 0.001) for diagnostic failure. Age, sex, pulmonary function, location of the lesion, CT findings of the lesion, longest diameter of the lesion, number of specimens, and length of aerated lung traversed by the needle did not differ significantly between the two groups.

## 4. Discussion

This retrospective study showed that CT-guided PTNB can be safely and effectively performed by an interventional pulmonologist to accurately diagnose a lung lesion. The sensitivity, specificity, PPV, NPV, and diagnostic accuracy of CT-guided PTNB were 96.1% (171/178), 100% (46/46), 100% (171/171), 86.8% (46/53), and 96.9% (217/224), respectively. The complications of the procedure were comparable to those reported in previous studies performed by interventional radiologists [11,16,17,18].

Several studies have assessed the feasibility and safety of ultrasound-assisted transthoracic biopsy when performed by a pulmonologist [12,13,14,15]. The diagnostic efficacy and safety of CT-guided PTNB using a laser guidance system, performed by a pulmonologist with 2 years of experience in CT-guided PTNB, have also been demonstrated [19]. However, the safety and outcome of CT-guided PTNB performed by an inexperienced pulmonologist had not previously been determined.

Table 6 summarizes the diagnostic performance of CT-guided PTNB reported in previous studies. The diagnostic yield in our study was comparable to that reported in previous studies in which the procedure was performed by interventional radiologists [1,20,21,22,23,24].

The rates of pneumothorax and pneumothorax requiring chest tube drain insertion (27.0% and 6.5%, respectively) were comparable to those previously reported (15.4–42.0% and 6–12.0%, respectively) after CT-guided PTNB [25,26,27].

Diagnostic failure in this study consisted of four technical failures and three false-negatives. The only significant risk factor associated with diagnostic failure was pneumothorax requiring chest tube insertion (odds ratio, 25.0; *p* < 0.001). Patients with chronic obstructive lung disease or emphysema are highly susceptible to developing pneumothorax during CT-guided PTNB [28,29,30]. As this complicates targeting the lung lesion and may require abandoning the procedure, the result in such cases may be diagnostic failure. Unlike previous studies, we did not find an association between diagnostic failure and male sex, lower lobe location, number of specimens, or a final diagnosis of malignancy [19,23].

Our study demonstrates that PTNB can be performed accurately and safely by a pulmonologist, without the need for an interventional radiologist. The implications of this finding are as follows. First, a pulmonologist can consistently assess patients and decide upon the need for an invasive procedure at the appropriate time. For patients, it means that they interact with only one physician. Scheduling also becomes less complicated, both for the patient and the physician. In addition, PTNB can be performed by interventional pulmonologists in centers without the support of interventional radiologists, as is often the case in the general hospitals of South Korea.

Although the interventional pulmonologist who participated in this study did not receive formal training in CT-guided PTNB, the diagnostic accuracy and safety profile were not different from those of an interventional radiologist. These encouraging results can be explained by several factors, as follows. The pulmonologist had 3 years of experience in many other respiratory procedures, including bronchoscopy (>1000 cases), endobronchial ultrasound (EBUS)-guided transbronchial needle aspiration (>200 cases), and ultrasound-guided thoracentesis (>200 cases), and therefore was thoroughly familiar with the anatomy of the chest and pleura. The pulmonologist also intensively studied the previously published literature, reviewing technical aspects and potential complications. Furthermore, in the early stages of PTNB, the pulmonologist frequently asked the opinion of an interventional radiologist from another university hospital by e-mail or phone. The interventional radiologist provided a considerable amount of advice about the procedure, which eventually allowed the pulmonologist to do well on his own.

Peripheral pulmonary lesions can be approached by bronchoscopy using radial EBUS in some cases. Recent meta-analysis [31] revealed that CT-guided PTNB was superior to radial EBUS for the evaluation of small peripheral pulmonary lesions (92%, 95% confidence interval: 88–95 vs 66%, 95% confidence interval: 55–76). However, for pulmonary lesions greater than 2 cm, the bronchoscopy using radial EBUS revealed a diagnostic yield of 80% and low complication rates compared with CT-guided PTNB. Further studies are needed to identify a proper individualized biopsy procedure.

There were several limitations to our study. First, because it was a retrospective review of performed procedures, there might have been selection bias. However, the pulmonologist performed CT-guided PTNB as part of his everyday routine practice, not just in selected patients. He refused to perform the procedure only when there was a particular reason, which was discussed with and confirmed by the interventional radiologist from the other university hospital. Second, although our patients were followed-up for at least 12 months, there were 24 who nonetheless still had indeterminate nodules. Though most of the previously published articles excluded indeterminate nodules to calculate diagnostic yield, this might result in a very high diagnostic yield in this study. Third, although the physician who performed the biopsy was not highly trained in CT-guided PTNB, he had 3 years of experience in other invasive procedures used in the diagnosis of suspected thoracic malignancies. Furthermore, all cases of CT-guided PTNB were done by only one pulmonologist in this study. Thus, our results cannot be generalized to pulmonologists with no experience in invasive procedures in the chest. Further studies performed by multiple pulmonologists with different levels of experiences are needed to confirm the conclusion of this study. However, given the high diagnostic yield of CT-guided PTNB and the acceptable rates of complications, our study shows that CT-guided PTNB can be performed by a pulmonologist in clinical settings without the support of an interventional radiologist. Moreover, our results demonstrate the growing importance and novel role of the interventional pulmonologists in diagnosing peripheral lung lesions.

## 5. Conclusions

In conclusion, when performed by a pulmonologist, CT-guided PTNB is a highly valuable and highly accurate procedure with acceptable complication rates. Thus, it can be performed by a pulmonologist in hospitals lacking the support of an interventional radiologist. Indeed, the results achieved in this study were similar to those of interventional radiologists. Pneumothorax requiring chest tube insertion was the only risk factor of diagnostic failure in our series.

## Figures and Tables

**Table 1:** Characteristics of patients undergoing computed tomography (CT)-guided percutaneous transthoracic needle biopsy (PTNB) (*n* = 248).

Characteristic	Value
**Patient**	
Age, years	68.2 ± 12.5
Male	174 (70.2%)
Pulmonary function test (*n* = 241), %	
FEV1	80.7 ± 20.1
FVC	79.8 ± 16.6
FEV1/FVC	71.9 ± 12.4
**Lung lesions**	
Location	
Upper lobe	147 (59.3%)
Middle lobe	9 (3.6%)
Lower lobe	92 (37.1%)
Computed tomography findings	
Solid	228 (91.9%)
Subsolid	17 (6.9%)
Ground-glass opacity	3 (1.2%)
Longest diameter of the lesion, mm	39.5 ± 19.3
Cavitary lesion	31 (12.5%)
**Procedure**	
Patient position during biopsy	
Supine	64 (25.8%)
Prone	141 (56.9%)
Decubitus	43 (17.3%)
Needle diameter (gauge)	
20	248 (100%)
Number of specimens (*n* = 244)	1.7 ± 0.7
Length of aerated lung traversed by needle, mm	14.5 ± 15.3
Emphysema along the needle pathway	30 (12.1%)
Poor cooperation	26 (10.5%)
Transfissural approach	13 (5.2%)

**Table 2:** Initial pathologic results and final diagnosis.

	Initial Pathologic Results (*n* = 244)	Final Diagnosis (*n* = 248)
**Malignant**	171	178
Primary lung cancer	165	172
Adenocarcinoma	90	93 ^a^
Squamous cell carcinoma	51	53 ^b^
NSCLC, NOS	7	8 ^c^
SCLC	11	12 ^d^
Large cell neuroendocrine carcinoma	1	1
Pleomorphic carcinoma	2	2
Malignant spindle cell tumor	3	2
Leiomyosarcoma	0	1 ^e^
Metastasis	6	6
Breast	2	2
Colon	1	1
Melanoma	1	1
Trachea	1	1
Salivary gland	1	1
**Benign**	69	46
Definite benign features	31	33
Pulmonary tuberculosis	13	14 ^f^
Organizing pneumonia	7	7
Tuberculous granuloma	6	6
Cryptococcosis	2	3 ^g^
PMF	1	1
Pulmonary infarction	1	1
GPA	1	1
Non-specific benign features	38	13
Indeterminate	4	24 ^h^

NSCLC, non-small-cell lung cancer; NOS, not otherwise specified, SCLC, small-cell lung cancer; PMF, progressive massive fibrosis; GPA, granulomatosis with polyangiitis. ^a^ Biopsy results included one false-negative result and two technical failures. ^b^ Biopsy results included one false-negative result and one technical failure. ^c^ Biopsy results included one false-negative result. ^d^ Biopsy results included one technical failure. ^e^ One malignant spindle cell tumor was surgically resected and proved to be leiomyosarcoma. ^f^ One lesion included non-specific benign features on PCNB, but the sputum study revealed pulmonary tuberculosis. ^g^ One lesion included indeterminate features on PCNB, but the surgically resected specimen revealed cryptococcosis. ^h^ Indeterminate lesions included the 24 lesions with non-specific benign features in patients with CT follow-up periods ≤12 months, refusal of re-biopsy, transfer to another hospital, and without outpatient follow-up.

**Table 3:** Diagnostic yield of CT-guided PTNB (*n* = 224).

Parameter	≤20 mm (*n* = 26)	21–39 mm (*n* = 99)	≥40 mm (*n* = 99)	Overall (*n* = 224)
True-positive, *n*	20	73	78	171
True-negative, *n*	5	22	19	46
False-positive, *n*	0	0	0	0
False-negative, *n*	0	2	1	3
Technical failure, *n*	1	2	1	4 ^a^
Sensitivity, %	95.2	94.8	97.5	96.1
Specificity, %	100.0	100.0	100.0	100
PPV, %	100.0	100.0	100.0	100
NPV, %	83.3	84.6	90.5	86.8
Diagnostic accuracy, %	96.2	96.0	98.0	96.9

PPV, positive predictive value; NPV, negative predictive value. ^a^ In the statistical analyses, technical failure cases were included in the false-negative category.

**Table 4:** Complication of PTNB (*n* = 248).

Complication Type	*n*
Pneumothorax	67 (27.0%)
Oxygen therapy and close observation	51 (20.6%)
Chest tube insertion	16 (6.5%)
Hemoptysis	13 (5.2%)
Oxygen therapy and close observation	13 (5.2%)
Hemothorax	2 (0.8%)
Chest tube insertion	2 (0.8%)

**Table 5:** Results of univariate analyses of the risk factors associated with diagnostic failure.

Characteristic	Diagnostic Success ^a^ (*n* = 217)	Diagnostic Failure ^b^ (*n* = 7)	*p* Value
**Patient**			
Age, years	68.2 ± 12.5	70.6 ± 10.9	0.623
Male	154 (96.9%)	5 (3.1%)	1.000
Pulmonary function test (*n* = 241), %			
FEV1	81.2 ± 20.0	79.0 ± 19.0	0.779
FVC	80.0 ± 16.8	81.3 ± 14.2	0.840
FEV1/FVC	72.2 ± 12.0	69.7 ± 18.2	0.604
**Lung lesions**			
Final diagnosis			
Malignancy	171 (96.1%)	7 (3.9%)	0.349
Benign	46 (100.0%)	0 (0%)	
Locations			
Upper lobe	127 (96.9%)	4 (3.1%)	0.851
Middle lobe	9 (100.0%)	0 (0%)	
Lower lobe	81 (96.4%)	3 (3.6%)	
CT findings			
Solid	201 (96.6%)	7 (3.4%)	0.496
Subsolid	13 (100.0%)	0 (0%)	
GGO	3 (100.0%)	0 (0%)	
Longest diameter of the lesion, mm	40.3 ± 19.5	31.6 ± 11.3	0.244
Cavitary lesion	27 (100.0%)	0 (0%)	1.000
**Procedure**			
Patient position during biopsy			
Supine	57 (98.3%)	1 (1.7%)	0.142
Prone	122 (97.6%)	3 (2.4%)	
Decubitus	38 (92.7%)	3 (7.3%)	
Number of specimens (*n* = 244)	1.7 ± 0.7	1.3 ± 0.6	0.402
Length of aerated lung traversed by needle, mm	14.7 ± 15.7	21.9 ± 16.9	0.236
Emphysema along the needle pathway	25 (92.6%)	2 (7.4%)	0.201
Poor cooperation	23 (92.0%)	2 (8.0%)	0.177
Transfissural approach	12 (100.0%)	0 (0%)	1.000
Aerated lung traversed by needle	141 (95.9%)	6 (4.1%)	0.427
**Complications**			
Pneumothorax	57 (93.4%)	4 (6.6%)	0.09
Pneumothorax, requiring chest tube insertion	11 (73.3%)	4 (26.7)	<0.001
Hemoptysis	12 (100.0%)	0 (0%)	1.000

CT, computed tomography; GGO, ground glass opacity. ^a^ True-positive and true-negative results. ^b^ False-positive, false-negative and technical failure results.

**Table 6:** Diagnostic performance of CT-guided biopsy reported in previous studies.

Studies	Country	No. of Nodules	Biopsy Methods	Sensitivity (%)	Specificity (%)	Diagnostic Accuracy (%)
Hiraki 2009 [1]	Japan	1000	Core biopsy	94.2	99.1	95.2
Yang 2015 [20]	China	311	Core biopsy	95.3	95.7	92.9
Wang 2016 [21]	China	1484	Core biopsy	94.4	100	94.8
Tian 2017 [22]	China	560	Core biopsy	92.0	98.6	94.6
Kim 2011 [24]	Korea	72	Aspiration	97.8	100	98.4
Choi 2013 [23]	Korea	153	Core biopsy	93.6	100	95.2
Ahn (present study)	Korea	224	Core biopsy	96.1	100	96.9
